# A Novel Endoscopic Method to Relieve Food Impaction Using an Inflatable Balloon

**DOI:** 10.1155/2015/357253

**Published:** 2015-07-21

**Authors:** Rohit Anand, Shashank Garg, Ethan Dubin, Sudhir Dutta

**Affiliations:** ^1^Johns Hopkins University-Sinai Hospital Program in Internal Medicine, Baltimore, MD 21215, USA; ^2^Division of Gastroenterology, Sinai Hospital of Baltimore, Baltimore, MD 21215, USA; ^3^University of Maryland School of Medicine, Baltimore, MD 21201, USA

## Abstract

Food impaction in the esophagus is a relatively common medical emergency. Most of these food impactions are relieved spontaneously. But for complete esophageal food impactions or impactions not relieved spontaneously, traditional endoscopic methods like using a Roth net, polypectomy snare, or rat or alligator tooth forceps are used to gently manipulate the food material into the stomach. However, these methods may not work in certain circumstances. We present a case of proximal esophageal food impaction that was relieved using an inflatable balloon after the conventional methods proved unsuccessful.

## 1. Introduction

Food impaction in the esophagus is one of the most common gastroenterology emergencies encountered. Of all the foreign body impactions in the esophagus in the adult population, meat impaction is the most common [1]. Most of these food boluses pass into the stomach without any intervention, but sometimes the impaction is not relived spontaneously. Usually patients that present with a food impaction have an underlying pathology in the esophagus that predisposes the ingested food to get stuck in the esophagus. In a study done by Kerlin et al., strictures and eosinophilic esophagitis were present in 35% and 33% of patients presenting with esophageal food impaction, respectively [2]. Adults usually present with dysphagia, inability to swallow saliva, neck tenderness, retrosternal fullness, choking, or blood stained saliva. Unfortunately, food impactions are not readily picked up on plain X-ray of the neck, chest, or abdomen. Barium studies should be avoided as the contrast can make it difficult to visualize the impaction on endoscopy and damage the endoscope. A CT scan can be done if one feels that the obstruction is incomplete but in a patient with high index of suspicion for a complete esophageal impaction, an urgent endoscopy is warranted. In patients that are unable to handle secretions, airway protection should be considered with an endotracheal intubation. Glucagon (IV) can be tried for esophageal dilation but endoscopic disimpaction of the food material is the treatment of choice due to its safety and efficacy [3]. An endoscopy to relive food impaction can be emergent, urgent, or nonurgent depending on the condition of patient, risk of complications, and size, shape, composition, and location of the food bolus. Even a nonurgent endoscopy should be done within 24 hours of a food bolus impaction because the major complication rate goes up by 14.1 times if the food bolus has been impacted for more than 24 hours [4]. Roth net, polypectomy snare, and rat or alligator tooth forceps are some of the commonly used devices used to relieve esophageal food impaction [5]. However, these methods may fail to relieve the impaction. We describe a case of proximal esophageal food impaction that was relieved using an inflatable through-the-scope (TTS) balloon.

## 2. Case

**Figure 1:**
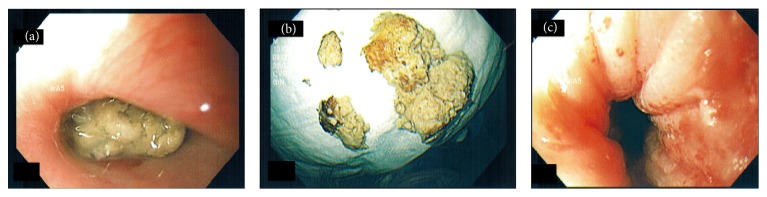
(a) Foreign body impacted in the proximal esophagus. (b) Foreign body after being removed from the esophagus. (c) Inflammation seen at the site of the removed foreign body in the esophagus.

**Figure 2:**
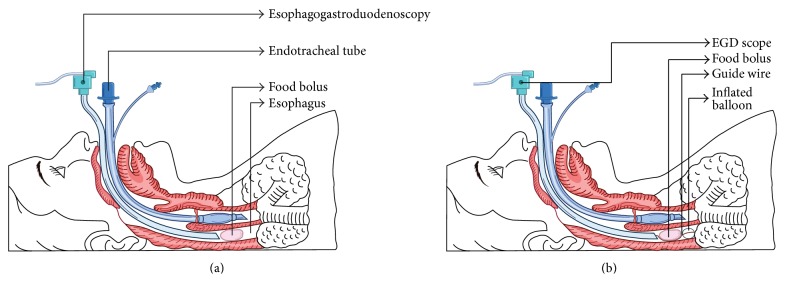
Schematic diagram showing the impacted foreign body in the proximal esophagus (a) and removal of the FB by inflating the balloon distal to it (b).

A 56-year-old man presented to the emergency department with productive cough, sensation of food stuck in his throat, and excessive drooling of saliva, following ingestion of a burger 3-4 hours ago. He had a past medical history of HIV and COPD. He smoked a pack of cigarettes a day for the last 25 years. On presentation to the emergency room, he was afebrile (temperature of 36.0°C) with stable blood pressure of 119/76 mmHg, but he was tachycardic up to 120 beats/minute. His lab work showed creatinine of 1.60 mg/dL, WBC count of 7,800/mm^3^, and hemoglobin of 13.3 g/dL. A complete food impaction was suspected and endotracheal intubation was performed to protect the airway. Upon entering the esophagus, a large piece of meat was seen impacted in the proximal esophagus, 18 cm from the incisors (Figure 1(a)). Repeated attempts to place a Roth net or basket around the piece of impacted meat were unsuccessful. Similarly, efforts to break the impacted meat with biopsy forceps and alligator forceps were unsuccessful. After trying the conventional methods to dislodge the meat bolus unsuccessfully for over 2 hours, a deflated TTS balloon (15–18 mm) was successfully passed along the side of the esophageal mucosa beyond the area of impaction (Figure 2(b)). The balloon was inflated to 18 mm caudal to the impacted food and the piece of meat was dislodged with gentle traction and was pulled into the mouth from where it was removed with the alligator forceps (Figure 1(b)). The esophagus was examined after the removal of the impaction and mild erythema was noted at the site of the impaction but no luminal narrowing or stricture was noted (Figure 1(c)). Multiple biopsies were taken from the proximal and distal esophagus to rule out eosinophilic esophagitis, amongst other pathologies. The patient was successfully extubated and discharged home. The biopsy results were negative for any esophagitis, metaplasia, dysplasia, or carcinoma. The impaction was thought to have happened from ingesting a very large intact piece of meat.

## 3. Discussion

Endoscopic disimpaction is the treatment of choice for food impaction. Conventionally it is performed endoscopically by food extraction or gentle manipulation into the stomach, either en bloc or piecemeal. Both rigid and flexible endoscope can be used to retrieve the food bolus. In a study by Gmeiner et al., it was seen that flexible endoscopy (FE) was successful in 93.4% of cases as compared to rigid endoscopy (RE) which was successful in 95.2% of the cases. The same study also showed that the major complication rate with FE was 0.00%, while with RE the major complication rate (esophageal perforation) was 3.2% (*p* < 0.002) [6]. Endoscopic devices used commonly for this purpose include Roth net, polypectomy snares, dormia basket, rat or alligator tooth forceps, or banding caps [2]. The conventional methods are successful most of the time but sometimes, like in our case, they fail to relieve the food impaction. The time to disimpact the food bolus in a piecemeal fashion should be closely monitored because the longer the food remains impacted, the higher the risk of esophageal perforation is. In our case we tried the traditional methods to remove the food bolus for over 2 hours but we could not disimpact it. As a result we used this method of relieving food impaction in cervical esophagus by inflating a TTS balloon distal to the impacted food item and removing it en bloc. Pushing the food through the entire length of esophagus would have been much more difficult and have a greater chance of perforation than pulling it into the mouth where we can visualize and monitor the motion of the food bolus. A similar method has been described for retrieving disk batteries impacted in the esophagus of children, where a through-the-scope balloon can be passed distally to the battery and then the balloon and the battery can be removed as a unit [1]. To date, this is the first reported case of this technique being used for food impaction. It is worth noting that endotracheal intubation is necessary before such a maneuver is attempted to prevent aspiration of the impacted food material.

In conclusion, TTS balloons can be used to relieve proximal esophageal food impaction when conventional methods have failed and the airway has been secured.
